# 

**Published:** 1838-01-03

**Authors:** 


### INDEX TO VOLUME I.—1838.

Abnormal Fœtus, cases of, 72, 269.
Abortion, Boivin on, 359.
Academy, Royal, of Paris, Analysis of memoirs of, 15.
Accidents, admitted into the Pennsylvania Hospital, fortnightly accounts of, 14, 38, 56, 71, 87, 102, 117, 136, 152, 168, 183, 198, 216, 232, 243, 258, 275, 295, 305, 328, 340, 357.
Acetate of lead, in cholera, remarks on, by Dr. Graves, 40; by Dr. Harlan, 187; in typhoid fever, 117.
Acids, mineral, poisonous effects of, 262.
Adhesive plaster, Liston's, 72.
Alibert, Baron, death of, 40; sketch of, 46.
Alkalies, poisonous effects of, 262.
Amaurosis, cauterization of the eye in, 120.
Ammoniaco-nitrate of silver, as a test of arsenic, observations on, 310.
Amputation, cases of, at the hip-joint, 39, 296; of the leg, 71, 327; of the thigh, 101, 152, 391; of the shoulder-joint, 167; of the arm, 168, 197.
Amputations, statistical accounts of, 103, 290.
Amussat, M. on the twisting of arteries, 136.
Anchylosis, new treatment in, 103.
Anencephalous fœtus, account of, 60.
Aneurism by anastomosis, operation for, 76.
Antimony, remarks on, 263.
Arseniate of potash, poisoning with, cases of, 40.
Arsenic, poisoning with, cases of, 259; antidotes for, 262, 328; iodide of, 408.
Arthritis, lectures on, 71, 88, 104, 121.
Atresia vaginæ, cases of, 46, 104.
Auriscope, account of an, 342.
Autoplasty, cases of, 14.
Autopsy of Joseph Clark, 135; of Leah Smith, 326.
Balsam copaiba, for chilblains, notices of, 77, 296.
Bafureira, notice of the, 184.
Bandages, Cutler on, notice of, 296.
Barton, Dr. J. Rhea, new treatment in anchylosis by, 103; bibliographical notice by, 112; on injuries of the wrist, 365.
Baths, hydro-sulphuretted, composition of, 136.
Bell, Sir C.'s surgery, bibliographical notices of, 77, 222.
Bridges, Dr. R. (see Journal of Pharmacy.)
Burns, treatment of, 200.
Cæsarian section, cases of, 15, 79. 118.
Cainca, properties of, 184.
Calculi, urinary, 248.
Calomel, in ophthalmia, 118.
Camphor, remarks on, 264; Hope's mixture, remarks on, 285.
Cancer, superficial, remarks on, 15; lecture on, 29.
Canton, American hospital at, 136.
Capsicum in dysentery, 343.
Carbonic acid, practical remarks on, 263.
Carson, Professor, (see Journal of Pharmacy;) on codeia, 415.
Catamenial irregularities in young women, 312.
Cauterization of the eye, 120.
Chapman Professor, lectures by, on chronic laryngitis, 3, 17; on acute laryngitis, 41, 57; on arthritis or gout, 72, 89, 105, 121; on acute rheumatism, 137, 153, 409; on variola or small-pox, 169, 185, 201, 217, 233; on varicella, 249; on variola vaccinæ, 266, 281; on the varioloid disease, 297, 313, 329; on rubeola, 345, 361; on scarlatina, 377, 393.
Chilblains, remarks on, 76.
Chloride of soda, in typhoid fever, 15.
Chloride of lime, in wounds, 200.
Chlorine, poisonous effects of, 261.
Cholera, malignant, acetate of lead in, 40, 187.
Chopart's method of amputating the foot, 422.
Civiale on urinary calculi, 248.
Clark, Sir James, promotion of, 72.
Clavicle, fractures of, apparatus for treating, 108; cases of dislocated, 152, 309.
Clinical lectures, (see Coates, Gibson, Harris, Harlan, Jackson, and Randolph.)
Clinical medicine, lectures on, by W. W. Gerhard, M. D. (see Gerhard.)
Club-foot, operation for the relief of, by Dr. Detmold, 198; by Dr. Dickson, 296.
Coates, Dr. B. H., lecture by, on the Thompsonian remedies, 64; operation by, for aneurism by anastomosis, 76; Dr. R., Popular Medicine by, bibliographical notice of, 173.
Cocks's surgery, bibliographical notice of, 61.
Colchicum, in rheumatic gout, 358.
Cooper, Sir A., on spermatocele, 279.
Copper, remarks on poisonous properties of, 264.
Cotton, raw, in erysipelas, 120.
Crania Americana, notice of, 113.
Creosote, mode of obtaining, 248.
Cummins, Dr., on dislocation of the femur, 278.
Cutler on bandages, notice of, 296.
Cynanche, laryngea, lectures on, 3, 17, 41, 57.
Cystoplasty, operation of, 264.
Davis, Dr. E. G., 117, 112, 286.
Delirium Tremens, lectures on, 212, 213.
D'Espine, on Orchitis, bibliographical notices of, 23, 43.
D'Etiolle, Leroy, on lithotripsy, 112.
Detmold, Dr., operation for club-foot by, 198.
Dickson, Dr., operation for club-foot by, 296.
Digitalis in delirium tremens, 118.
Dilatation and contraction of pupil after death, 360.
Discharges from Pennsylvania Hospital, 357, 375, 390, 403, 418.
Dislocation, cases of, of both thighs, 39; of the knee, 40, 72; of radius backward, 197; of thigh, 278, 305; of shoulder, 55, 70, 310, 340, 419; of clavicle, 152; of metatarsal bones, 424.
Drake, Dr., elements of pathology by, notice of, 189.
Dropsy, clinical lectures on, 34, 371.
Duges, M., death of, 392.
Dunglison, Dr., 198, 376.
Dysentery, clinical lectures on, 239, 256; treatment of, 285, 343, 347, 392.
Ear, Kramer on the, bibliographical notices of, 204, 219.
Eberle, Dr., death of, 53.
Encephalon, diseases of, by Andral, bibliographical notice of, 352.
Enlargement of the veins of the scrotum, mode of relieving, 360.
Epilepsy, lecture on, 93.
Erectile tumours, Dr. Pancoast's successful operation for, 92.
Erysipelas, on the treatment of, 120.
Esquirol on insanity, bibliographical notice of, 254.
Eve, Professor Paul F., cases of amputation of the lower joint, 306; of division of adductor longus femoris, 406.
Exstrophy, congenital, of the bladder, case of, 16.
Extirpation of parotid gland, 126; of the uterus, 200.
Fistula in ano, lecture on, 47.
Fœtus, anencephalous, case of, 60; abnormal, cases of, 72, 269.
Fractures, statistics of, 24; new treatment for, 343, 376; of the skull, case of, 77; ununited, 151; comminuted, of leg, 242, 274; compound of elbow joint, 294; compound of the thigh, 405.
Fungus hæmatodes, case of, 101.
Gastritis, lecture on, 238.
Gerhard, Dr., clinical lectures by, on typhus and typhoid fever, 10; introductory to a course, 146; on acute rheumatism, 147; on rubeola, 162; on serous membranes, 175; on tubercular meningitis, 179; on acute meningitis, 189; on chronic meningitis, &c., 193; on apoplexy, 209; on delirium tremens, 212; on diseases of the spinal marrow, 224; on mucous membranes, 227; on gastritis, 235; on dysentery, 239, 256; on laryngitis, 270, 300; on bronchitis, 302; on diseases of the heart, 318, 336, 354, 371; on pleurisy and pericarditis, 399.
Gibson, Professor, clinical lectures, on bony tumours, 9; on caries of the spine, 32; on sloughing ulcers, 52; on ulcers of the lip, 66; reply to Dr. Harlan's strictures, 88; on entropium, phymosis, &c., 412; Dr. C. B., appointment of, 419.
Glizen, Dr., on dysentery, 392.
Goddard, Dr., account of a double uterus by, 19; autopsy by, 135.
Gonorrhœa, Dr. Ricord's practice in, 183.
Gout, (see Arthritis.)
Granville, Dr., on counter irritation, bibliograph. notice of, 381.
Gunpowder, on the medicinal use of, 126.
Halford, Sir Henry, sketch of, 208.
Hall, Dr. J. C., case of pelvic tumour by, 268.
Hare-lip, operations for, 13, 102.
Harlan, Dr., clinical lectures by, on bony tumours, 85; on ophthalmia, 86; on syphilis, 99, 100; remarks on acetate of lead in cholera, by, 187.
Harris, Dr. T., lectures by, on wounds of nerves, 1; on stricture of the rectum, 79; on hydrocele, 50; on stricture of the urethra, 131; on diseases of the bones, 158; on ulcers, 165; on poisoned wounds, 250; on tetanus, 231; reduction of dislocations by, 55, 70; amputations by, 71, 152; operation for varicose veins by, 89; double hare lip, 102; phymosis, 102.
Hayward, Dr., report by, 341.
Health, Dr. Southwood Smith's philosophy of, bibliographical notice of, 108; Dr. Bell on, do., 333.
Hemlock baths, in cutaneous diseases, 423.
Hernia cerebri, case of, 292.
Heurteloup, new work on lithotripsy by, 155.
Holmes, Dr., Prize Essays by, bibliographical notice of, 198.
Homœopathy, Dr. Jeanes on, bibliographical notice of, 332.
Hope's camphor mixture, notice of, 285.
Hosack's Practice of Physic, bibliographical notice of, 398.
Hospitals, Pennsylvania, reports from, 13, 20, 38, 55, 70, 87, 101, 116, 136, 151, 167, 184, 197, 216, 231, 247, 258, 271, 292, 305, 326, 340, 357, 375, 390, 403, 421; lectures at, (see lectures;) Philadelphia, reports from, 135, 327; lectures at, (see lectures.)
Hotel Dieu, notice of, 343.
Hydrocyanic acid, on the poisonous effects of, 264.
Hydrated per-oxide of iron, 328.
Hydrocele, lecture on, 50.
Hydro-sulphuretted baths, 136.
Hysterotomy, case of, 15.
Hulme, Dr., death of, 39.
Huntt, Dr. Henry, biographical sketch of, 363.
Incised wound of abdomen, case of, 242.

Inflammation of the stomach and intestines, from pork, case of, 5; of the eyes, external use of calomel in, 118: of the serous membrane, Dr. Gerhard on, 175; of the brain, do. 215; of the mucous membrane, do., 227; of the testicle, cured by compression, 278; acute, of membranes of the heart, Dr. Gerhard on, 318; of the heart, do., 336.
Injection of vessels of the umbilical cord in retained placenta, 119.
Injuries, primary treatment of, Stevens' clinical lecture on, 28.
Insane Hospital for state poor, 399.
Institutes of Surgery, Sir Charles Bell's, bibliographical notice of, 77, 222.
Intestine, wounds of, Jobert's method of treating, 71.
Iodine, poisonous effects of, 262.
Ives, Dr., death of, 63.
Jackson, J. B. S. Dr., case of malformed female genital organs, by, 270.
Jackson, Dr., on typhoid fever, bibliographical notice of, 387.
Jackson, Dr. James, jr., notice of, 15.
Jackson, Professor, clinical lectures by, on dropsy, 34; on neuralgia, 53, 65, 68, 83; on epilepsy, 93.
Jaw-bone, lower, amputation of, 306.
Jenner, Dr., notice of the life of, 383.
Johnston, Dr., on syphilis, 348.
Journals, Louisville Medical, 174; American of Pharmacy, 204; Southern Medical, 343.
Kermes mineral, in pneumonia, 184.
Kramer, Dr., on the ear, bibliographical notice of, 204, 219.
Laryngitis, lectures on, 3, 17, 41, 59, 270, 300.
Larynx, case of rupture of, 151.
Lectures, (see Chapman, Coates, Gerhard, Gibson, Harlan, Harris, Jackson, Randolph.)
Libraries, American Medical, 8; Select Medical, 28, 306, 343.
Lisfranc, M., on cancers, 15.
Liston's Surgery, bibliog. notice of, 61; adhesive plaster, 72.
Lippincott, Dr., case of abnormal fœtus by, 269.
Lithotripsy, cases of, 14, 56, 116, 155, 159, 241.
Lithotomy, 125, 374.
Lobelia inflata, lecture on, 64.
Louis, Dr., on emphysema, bibliographical notice of, 111; observations by, on chronic peritonitis and double pleurisy, 333.
Luxation, (see dislocations.)
Macartney on inflammation, bibliographical notice of, 368.
Mackintosh, Dr., death of, 15.
Magnetism, animal, 424.
Malformation of genital organs, case of, 276.
Manganese, poisonous effects of, 278.
Marriage, remarks on, 72, 114; Quetelet on the effects of, 71.
McDowell, Dr., on slippery-elm bougies, 244.
Measles, lectures on, 162, 345, 361.
Meatus urinarius, instrument for dilatation of, 247.
Medical Colleges, 144, 243; of Georgia, 198; of Yale, 328; Convention, 198.
Meigs, Dr., on midwifery, bibliographical notice of, 46; remarks on dysentery by, 285; on croup, 397, 413; Dr. J. F., clinical reports by, 292, 305, 326, 340, 357.
Meningitis, lectures on, 179, 189, 193.
Mental disorders, Esquirol on, bibliographical notice of, 254.
Mercer, Dr., U. S. N., letter from, 331.
Mercury, poisonous effects of, 263, 278.
Miller, Dr. T., biographical notice by, 363.
Mineral acids, poisonous effects of, 262.
Morton's Crania Americana, notice of, 113.
Mortality in Philadelphia, 104, 243.
Mott, Dr., 38, 79.
Mucous membranes, inflammation of, lecture on, 227.
Muller's Physiology, bibliographical notice of, 286.
Naval service, on the examination of recruits for, 234.
Nerves, wounds of, lecture on, 1.
Neuralgia, lectures on, 53, 65, 68, 83.
Nitrate of potash, effects of poisoning by, 262.
Norris, Dr., operation by, for varicose veins, 151; statistics, by, 290.
Nuclei of urinary calculi, Civiale on, 244.
Opium, poisonous effects of, 277; adulteration of, 360.
Ophthalmia, Dr. Harlan on, 99.
Orchitis, D'Espine on, bibliographical notices of, 23, 43.
Organic irritants, 283.
Oxalic acid, poisonous effects of, 264.
Pancoast, Dr., account of operation for erectile tumour by, 92.
Parotid gland, extirpation of, 120.
Peace, Dr. E., appointment of, 405.
Pelvic tumour, case of, 268.
Pepper, Dr., case of poisoning from pork, by, 5.
Phymosis, case of, 102.
Physick, Dr., death of, 8.
Phosphorus, poisonous effects of, 262.
Pneumothorax, case of, 274.
Poisons, practical remarks on, 261.
Pork, case of poisoning from, 5.
Prize, Medical, Questions, 376, 419.
Quetelet, on Marriage, notice of, 62.
Randolph, Dr. J., lectures by, on cancer, 29; on fistula in ano, 47; on tumours, 114; on lithotripsy, 159; operations by, for hare-lip, 13; for lithotripsy, 116, 241; for excision of a tumour of the breast, 117; for amputation of the thigh, 101; at the shoulder-joint, 167, of the arm, 168, 197.
Rectum, stricture of, lectures on, 79.
Reid, Dr., on the eighth pair of nerves, bibliographical notices of, 128, 140.
Re-vaccination, results of, in Prussia, 237.
Rheumatisms, lectures on, 137, 147, 153, 409.
Ricord, Dr., on venereal diseases, bibliographical notices of, 143, 155; on the treatment of gonorrhœa, 183.
Ruschenberger, Dr., on chilblains, 70.
Rubeola, lectures on, 162, 345, 361.
Rufz, Dr., letter from, 317.
Rupture of the perineum in females, 423.
Ryan, Dr., on marriage, strictures on, 174.
Scapula, excision of the spine of, 158.
Scarlatina, lectures on, 377, 393.
Scirrhus, (see Cancer.)
Serous membranes, inflammations of, lecture on, 175.
Sharpless, Dr., on prolapsus uteri, 38; account of an auriscope by, 342.
Slippery-elm, bougies of, 244.
Small-pox, (see Variola.)
Smith, Dr. H. H., clinical reports by, 103, 151, 167, 184, 197, 216, 231, 241, 375, 390, 403, 418.
Smith, Dr. N. R., cases of lithotripsy reported by, 14; extirpation of the parotid gland by, 419.
Smith, Dr. Southwood, on the philosophy of health, bibliographical notice of, 108.
Smith, Dr. Thomas, election of, 419.
Society, Philadelphia Medical, election for officers of, 38.
Softening of the brain, lecture on, 193; of the spinal marrow, case of, 273.
Speculum, uteri, of glass, 119.
Spermatocele, Sir A. Cooper on, 279.
Spermatorrhœa, treatment of, 422.
Spine, caries of the, lecture on, 32.
Stevens on the primary treatment of injuries, bibliographical notice of, 28; on lithotomy, do., 125.
Stewardson, Dr., election of, 198.
Stones, Dr., case of compound fracture of the skull, by, 77.
Stricture of rectum, lecture on, 79; of urethra, lecture on, 131.
Strychnine, remarks on, 264; nitrate of, for paralysis, 376.
Sulphuretted hydrogen, poisonous effects of, 264.
Surgery, Liston's practical, and Cock's operative, bibliographical notice of, 61; Bell's institutes of, do., 77, 222.
Suture, intestinal, remarks on, 70.
Syphilides, Martins on, bibliographical notice of, 315.
Syphilis, lectures on, 99, 100; iodide of iron in, 344.
Syphilis, secondary, on the salts of silver in, 348.
Tartar emetic, in pneumonia, M. Chomel on, 408.
Teeth, wisdom, on abnormal growth of, 171.
Tendo-achillis, division of, 198.
Tetanus, lecture on, 321.
Thomson, Dr. R. D., on poisons, 261, 277.
Transactions of the N. Y. Medical Society, bibliographical notice of, 157.
Trudeau, Dr., on the abnormal growth of the wisdom teeth, 171; on the salts of silver in syphilis, 348.
True value of experience in medicine, 419.
Tumour erectile, operations for, 76, 92; caseous, excision of, 117; pelvic, case of, 268.
Tumours, Warren on, bibliog. notice of, 6; lecture on, 114.
Typhoid fever, lecture on, 11; chloride of soda in, 15; acetate of lead in, 117; Jackson on, bibliographical notice of, 287.
Typhus fever, lecture on, 10.
Twisting of arteries, M. Amussat on, 136.
Ulcers, lecture on, 165; of the lip, lecture on, 66.
Ulcer, sloughing, lecture on, 52; case of, 327.
Umbilical cord, injections into, 119.
University of Pennsylvania, graduates in, 155.
Uteri, prolapsus, Dr. Sharpless on, 38.
Uterus, extirpation of, 200; Duparque on the, bibliographical notice of, 221.
Uterus, double, account of a, 19.
Vaccination, lectures on, 265, 281.
Vaccine fluid, composition of, 360.
Vagitus uterinus, case of, 119.
Vallet's ferruginous pills, 421.
Varicella, lecture on, 249.
Varicocele, Dr. Fricke on, 120; Sir A. Cooper on, 279.
Varicose veins, operations for, 87, 151.
Variola, lectures on, 169, 185, 201, 217, 233.
Varioloid, lectures on, 297, 313, 329.
Vedder, Dr., clinical report by, 327.
Velpeau, M., on exstrophy of the bladder, 16; on burns, 200; on fractures, 343.
Wallace, Dr., statistical account of fractures by, 209; clinical reports by, 101, 274, 294.
Warren on tumours, bibliographical notice of, 6; Dr. J. Mason's case of autoplasty, 14; Dr. J. C. 306, 376.
Warrington, Dr., oration by, bibliographical notice of, 46; his translation of Duparque on the uterus, do., 221; on abnormal fœtus, 269.
West, Dr., case of anencephalous fœtus, by, 60.
Williamson, Dr., on the examination of naval recruits, 234.
Wistar, Dr. Jones, death of, 39.
Works announced, 419.
Wounds, case of incised, 242; of gunshot, 258; of lacerated, 295.
Wounds of nerves, lecture on, 1; poisoned, lecture on, 250.
Zinc, oxide of, case of poisoning by, 308.

### IMPROVED SURGICAL INSTRUMENTS.

*For the radical cure of Hernia or Rupture.*

It will be recollected that the Philadelphia Medical Society appointed a Committee of Surgeons in the year 1834, to examine the character of the various Trusses, and other proposed means of Radical Cure in Hernia.

The preliminary report of this Committee was read before the Society, on the 5th and 12th of December, 1835, and was published in the American Journal of the Medical Sciences.—This report gave to the instruments of Dr. Chase, the decided preference over all others, in the treatment of this disease.

Since the preliminary report was laid before the profession, members of this Committee have devoted an amount of time and attention to the subject of these instruments commensurate with the importance of the disease. Their researches into the method of employing them, their physiological action, and the mode in which they effect the cure of Hernia, engaged their attention until the 29th of April, 1837, when the fruit of their labors was laid before the Society in their final report, which was also accepted, and published in the above-named Journal.

The instruments of Dr. Chase, are five in number, viz:—The Inguinal, Ventro-Inguinal, Femoral, Umbilical and double Trusses. They are constructed upon purely Anatomical and Surgical principles, and each instrument can be adapted to the appropriate form of the disease only. By reference to the reports above mentioned, it will be seen, that these Instruments are now brought to such a degree of perfection, that they may be considered as reducing the cure of Hernia almost to a certainty, when judiciously employed.

They are adapted to all ages—to both sexes, and do not interfere with the ordinary avocation of the wearer.

It is the express desire of the inventor to place and retain these Instruments in the hands of medical men; for they are not designed, nor can they be employed with safety, except by Surgical scholars and practitioners.

*For the manner of using these Instruments*, (see, Chase on the Radical Cure of Hernia.) Any information respecting them will be freely given to the profession.

All communications of a professional nature, must be addressed (post paid) to the Inventor. Those of a business character, to Samuel C. Sheppard, General Agent, 107 South Ninth street, Philadelphia

### INELASTIC SUSPENSORY TRUSS.

*For the Relief and Cure of Cirsocele or Varicocele.*

This new and beautiful instrument has been recently constructed and introduced into practice, in this, and other cities by Dr. Chase. It is novel in construction, and acts, by supporting permanently, the parts implicated in this disease.

For a full description and drawing of this Instrument, with cases reported from its use, see appendix to the final report of the Committee on Hernia, with notes, etc—soon to be published by J. G. Auner, 331 Market Street. Suspensories to be had of Samuel C. Sheppard, 107 South 9th Street, Philadelphia.

### J. G. AUNER,

331 Market Street,

Will shortly publish the Final Report of the Committee of the Philadelphia Medical Society, on the construction of instruments, and their mode of action in the Radical Cure of Hernia or Rupture," from three years' observations; with notes, illustrations, and additional cases of Hernia, and diseases resembling Hernia: containing a tabular statement of two hundred cases of this disease. Also, illustrations of novel instruments, designed for the treatment of diseases affecting similar parts, By HEBER CHASE, M. D. Member of the Academy of Natural Sciences, Honorary Member of the Philadelphia Medical Society, etc.

### TERMS.

The Examiner is published every alternate Wednesday, on a super-royal sheet at *Three dollars* per annum, *in advance.* Each number contains sixteen octavo pages of double column, making an annual volume of upwards of four hundred pages, furnished with an index and title page.

Letters and communications may be addressed to the "Editors of the Medical Examiner," and must be *post paid, otherwise they will not be taken from the office.*

Orders for subscription, accompanied with a remittance, will be met by prompt attention

Persons enclosing *Five* dollars, will be entitled to *two copies.*

Advertisements are inserted at the rate of $1 for 12 lines.

Office No. 230 Spruce street, between Sixth and Seventh streets.

